# Green synthesis of silver tungstate/ionic liquid-modified electrode for highly efficient electrochemical detection of the antidepressant Vortioxetine

**DOI:** 10.1038/s41598-025-28420-9

**Published:** 2025-12-05

**Authors:** Sahar Zinatloo-Ajabshir, Hamid Akbari Javar, Hadi Mahmoudi-Moghaddam, Ali Azari

**Affiliations:** 1 Department of Chemical Engineering, University of Bonab, Bonab, P.O. Box. 5551395133, Iran; 2 Department of Pharmaceutics, Faculty of Pharmacy, Tehran University of Medical Sciences, Tehran, Iran; 3 Pharmaceutics Research Center, Institute of Pharmaceutical Sciences, Kerman University of Medical Sciences, Kerman, Iran; 4 Research Center for Environmental Pollutants, Qom University of Medical Sciences, Qom, Iran; 5 Department of Environmental Health Engineering, School of Health, Qom University of Medical Sciences, Qom, Iran

**Keywords:** Antidepressant, Vortioxetine, Ionic liquid, Sensor, Voltammetry, Nanocomposite, Chemistry, Materials science, Nanoscience and technology

## Abstract

In this experimental work, silver tungstate nanostructures were fabricated by a simple combustion synthesis method utilizing sucrose molecules as a new environmentally friendly fuel and structure-controlling agent. Subsequently, a carbon paste electrode (CPE) was modified with the Ag_2_WO₄ nanocomposite and an ionic liquid (IL) to exploit the synergistic effects of both materials, yielding a cost-effective and highly efficient platform for the electrochemical detection of Vortioxetine (VRT). The Ag_2_WO₄/IL-modified CPE exhibited markedly enhanced electrochemical activity compared to the bare electrode, as evidenced by the increased oxidation currents and reduced charge-transfer resistance observed in cyclic voltammetry and electrochemical impedance spectroscopy analyses. The sensor demonstrated excellent linearity across a wide concentration range (0.03–60 µM) under optimized conditions, with a detection limit of 0.01 µM, indicating high sensitivity in adsorptive differential pulse voltammetry (AdsDPV) measurements. Additionally, the platform displayed excellent selectivity, stability, and reproducibility, with recovery values between 97.0 and 103.3% and relative standard deviation (RSD) values from 2.1 to 3.6%. The findings affirm the potential of the Ag_2_WO₄/IL/CPE sensor for precise quantification of VRT in biological fluids and pharmaceutical formulations. This work presents a promising electrochemical sensing strategy with potential extensions to other neuroactive drugs in clinical diagnostics.

**Supplementary Information:**

The online version contains supplementary material available at 10.1038/s41598-025-28420-9.

## Introduction

Vortioxetine, a novel antidepressant with multimodal activity, has received FDA approval for the clinical management of major depressive disorder (MDD). Vortioxetine, unlike traditional antidepressants used for MDD, exhibits a unique multimodal mechanism of action and a distinctive clinical profile, supporting its potential use as a first-line therapy or as an alternative for patients with inadequate response to conventional treatments^1,2^. Preclinical studies suggest that vortioxetine may exert its antidepressant effects by modulating neurotransmission across multiple systems, including dopamine, serotonin, acetylcholine, norepinephrine, histamine, gamma-aminobutyric acid, and glutamate^3,4^. Severe adverse effects such as pancreatitis, rapid blood pressure elevation, and suicide attempts have been reported for VRT. These events correspond with increased levels of serotonin and additional neurotransmitters in the central nervous system, contributing to their modulatory effects^5,6^. Therefore, monitoring vortioxetine concentrations in pharmaceutical formulations and biological fluids is crucial to ensure patient safety.

Various analytical methods, including liquid chromatography coupled with multiple detection systems, have been developed for the quantification of vortioxetine in diverse sample matrices^7,8^. Nevertheless, these techniques tend to be costly, time-intensive, and frequently constrained by limited portability^9,10^. Due to their portability, simplicity, and high sensitivity, electrochemical methods have attracted considerable interest for practical applications. These techniques offer advantages such as low cost, relatively fast analysis times, and the ability to perform in situ detection, setting them apart from other approaches^11–13^.

Recently, nanotechnology has been successfully employed to develop various nanomaterials with novel properties and applications across numerous fields^14–16^. For instance, carbons, metals and their oxides are notable nanomaterials characterized by unique nanostructures and distinct advantages, including exceptional electrocatalytic activity, excellent conductivity, large specific surface area, enhanced signal response, and pronounced overpotentials, particularly when utilized in electrochemical nanoscale sensor architectures^17–19^.

Silver tungstate (Ag₂WO₄) is emerging as a candidate sensing material because it combines semiconductor redox activity with a rich surface chemistry^20^. Ag₂WO₄ has unique chemical and physical properties arising from its complex Ag–W coordination framework, and has been intensively studied for diverse applications, including electrochemical sensing^21^. The integration of Ag⁺ and WO₄^2^⁻ units in Ag₂WO₄ generates a synergistic system where the wide-bandgap semiconducting framework of tungstate is electronically modified by the presence of silver ions. While WO₄^2^⁻ provides redox-active centers and structural stability, Ag⁺ contributes to enhanced charge transfer by introducing intermediate states within the bandgap^22,23^. This combination results in a narrowed effective bandgap (2.6–3.1 eV), improved conductivity, and a high density of electroactive sites-features that are critical for achieving high-performance electrochemical sensing^24^. The synergistic interplay between the Ag and W centers is a key factor in the electrocatalytic activity and sensor responsiveness of Ag₂WO₄-based.

Ionic liquids (ILs) have gained considerable interest recently because of their exceptional features, including wide electrochemical windows, catalytic properties, negligible vapor pressures, high conductivity, and robust chemical and thermal stability^25^. These unique characteristics have enabled ILs to be used both as binders in the fabrication of carbon ionic liquid electrodes and as modifiers on electrode surfaces to enhance their performance^26^.

In recent years, the exploitation of green chemistry-based synthesis approaches has attracted much interest from researchers owing to their high potential to diminish the toxicity of nanostructures^27^. Studies have demonstrated that the usage of toxic and non-beneficial chemical reagents for human health and the environment can significantly affect the toxicity of nanostructures and result in undesirable and unintended health effects^28^. In this study, sucrose molecules were employed as a fuel and a novel, environmentally friendly structure-controlling agent to fabricate silver tungstate nanostructures for the first time. A simple and rapid auto-combustion approach is utilized to produce nanoscale silver tungstate. The resulting Ag₂WO₄ nanomaterial was employed to modify an electrode, which was further enhanced with an ionic liquid to develop a sensitive electrochemical sensor for the pharmaceutical drug Vortioxetine. The incorporation of Ag₂WO₄ markedly enhanced the sensor’s sensitivity. Finally, Ag_2_WO₄/IL-modified CPE was successfully employed to detect Vortioxetine in urine and tablet, highlighting its potential for practical applications in bioanalysis and drug monitoring.

## Experimental

### Chemicals and materials

The Vortioxetine (> 98%), silver nitrate (AgNO₃, 99.9%), sucrose (> 99.5%), and sodium tungstate dihydrate (Na₂WO₄·2H₂O, 99%) were purchased from Sigma-Aldrich and used without further purification. The ionic liquid N-butyl pyridinium hexafluorophosphate (BPPF₆, 99%) was obtained from Merck. All standard solutions were prepared using deionized water.

Using an Autolab potentiostat/galvanostat 204, electrochemical measurements were performed within a three-electrode system. The electrochemical cell consisted of an Ag/AgCl reference electrode, a platinum wire counter electrode, and either a bare carbon paste electrode (CPE) or an Ag₂WO₄/ionic liquid-modified CPE (Ag₂WO₄/IL/CPE) as the working electrode. The working CPE had a diameter of 3.0 mm, corresponding to a geometrical area of approximately 0.067 cm^2^. Investigation of surface morphology features and determination of elemental composition of as-produced silver tungstate powders were done utilizing FESEM (Zeiss Sigma 300) as well as a Philips CM30 TEM. The analysis of XRD with a diffractometer of Philips Company was performed to identify the crystalline structure and phase of as-prepared silver tungstate.

### Sucrose-assisted procedure for fabrication of nanoscale silver tungstate

First, in one beaker, 2 mM of silver nitrate was dissolved in 10 mL of deionized water. In another beaker, 1 mM of sodium tungstate dihydrate and 0.5 mmol of sucrose were dissolved in 15 mL of deionized water. Next, the contents of the second beaker were added dropwise to the first beaker (while stirring on a magnetic stirrer). The resulting mixture was poured into a crucible and transferred to an electric furnace, and its temperature was enhanced to about 330 °C^29^. In less than 15 minutes, the resulting mixture first began to dehydrate and then ignited. The white powder resulting from complete combustion was utilized for further investigations and characterization of its properties.

### Sensor fabrication

A homogeneous carbon paste was prepared by manually mixing 0.05 g of Ag₂WO₄ nanoparticles, 0.45 g of graphite powder, 0.3 mL of ionic liquid (IL), and 0.8 mL of mineral oil thoroughly in a mortar. The prepared paste was carefully packed into the end of a glass tube, and a copper wire was inserted to establish electrical contact. Prior to each measurement, excess fresh paste was extruded from the tube, and the electrode surface was carefully smoothed using glossy weighing paper to ensure a clean and reproducible sensing surface.

### Real sample preparation

To prepare a Vortioxetine solution, five 10 mg Vortioxetine tablets were powdered, and a certain amount of the drug was accurately weighed. The powder was dissolved in 0.1 M phosphate-buffered solution (PBS) (pH 7), sonicated, filtered to remove excipients, and diluted to 25 mL. The solution was used fresh or stored at 4 °C for up to 24 h.

A urine sample collected from one of the co-authors, who is a healthy individual, was diluted using 0.1 M phosphate buffer (pH 7.0) and centrifuged at 4000 rpm for 10 min to remove suspended solids and impurities. The supernatant was then filtered through a filter paper to further eliminate any remaining solids. Known concentrations of Vortioxetine were then added to the pre-treated urine samples.

### Ethics statement

This study was conducted in accordance with the ethical principles of the Declaration of Helsinki and the national guidelines for research ethics. It involved the analysis of a single, non-identifiable urine sample obtained from one of the co-authors. The donor provided written informed consent for the use of this biological material in the study. The study was internally reviewed by the research team and was deemed exempt from formal ethics committee review by the Institutional Ethics Committee of Kerman University of Medical Sciences, as the sample was sourced from a co-author and all data were handled anonymously, involving no vulnerable populations. All procedures were carried out in compliance with relevant institutional and international guidelines and regulations.

## Results and discussion

### Characterization of nanostructure

**Fig. 1:**
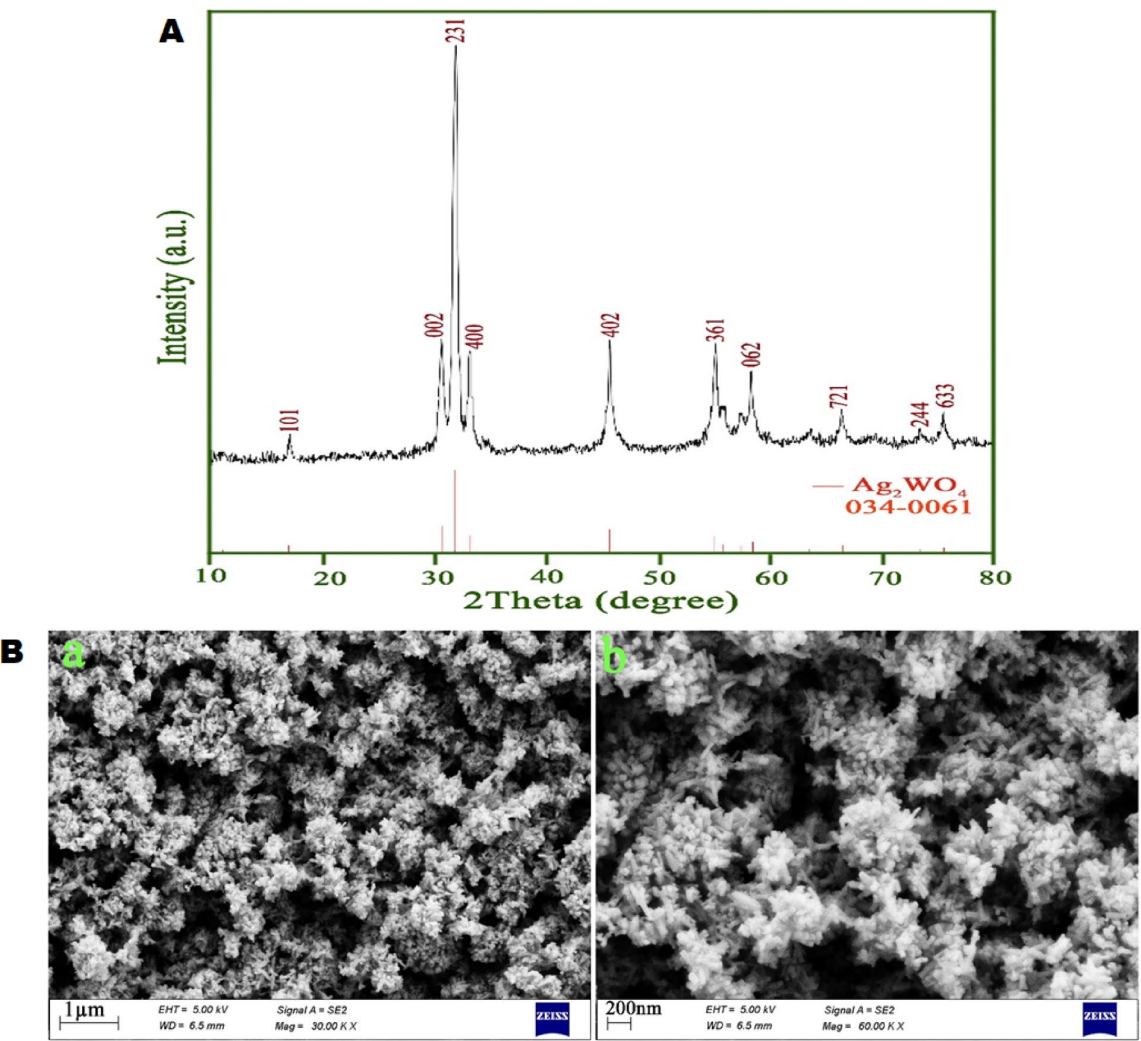
(**A**) X-ray diffraction (XRD) pattern and (**B**) FESEM images at different magnifications of the silver tungstate sample prepared via combustion approach with the help of sucrose molecules.

In order to benefit from the principles of green chemistry, we applied sucrose molecules as a novel fuel via a fast and simple combustion approach to fabricate silver tungstate nanostructures. By looking closely at the XRD pattern, it can be seen that the synthesized sample only exhibits signals belonging to the orthorhombic phase of silver tungstate, which is in good agreement with JCPDS No. 034-0061 (Fig. 1A). The lack of detection of signals related to any type of impurity in the XRD pattern of the sample is evidence of the purity of the silver tungstate powder produced with the help of sucrose molecules. The crystal size of the as-synthesized silver tungstate powder was computed to be near 22 nm employing the Scherrer equation^30^.

In order to characterize the morphological features of the silver tungstate sample prepared by combustion approach with the help of sucrose molecules, FESEM images were taken at different magnifications, which can be seen in Fig. 1B. The formation of almost uniform nanoclusters, which are created by assembling nanoparticles together, is demonstrated in the images. It seems that sucrose molecules, in addition to being reducing agents, can play the role of a morphological engineering agent during synthesis, causing growth in specific directions and consequently creating cluster-like silver tungstate nanostructures^31^.

**Fig. 2:**
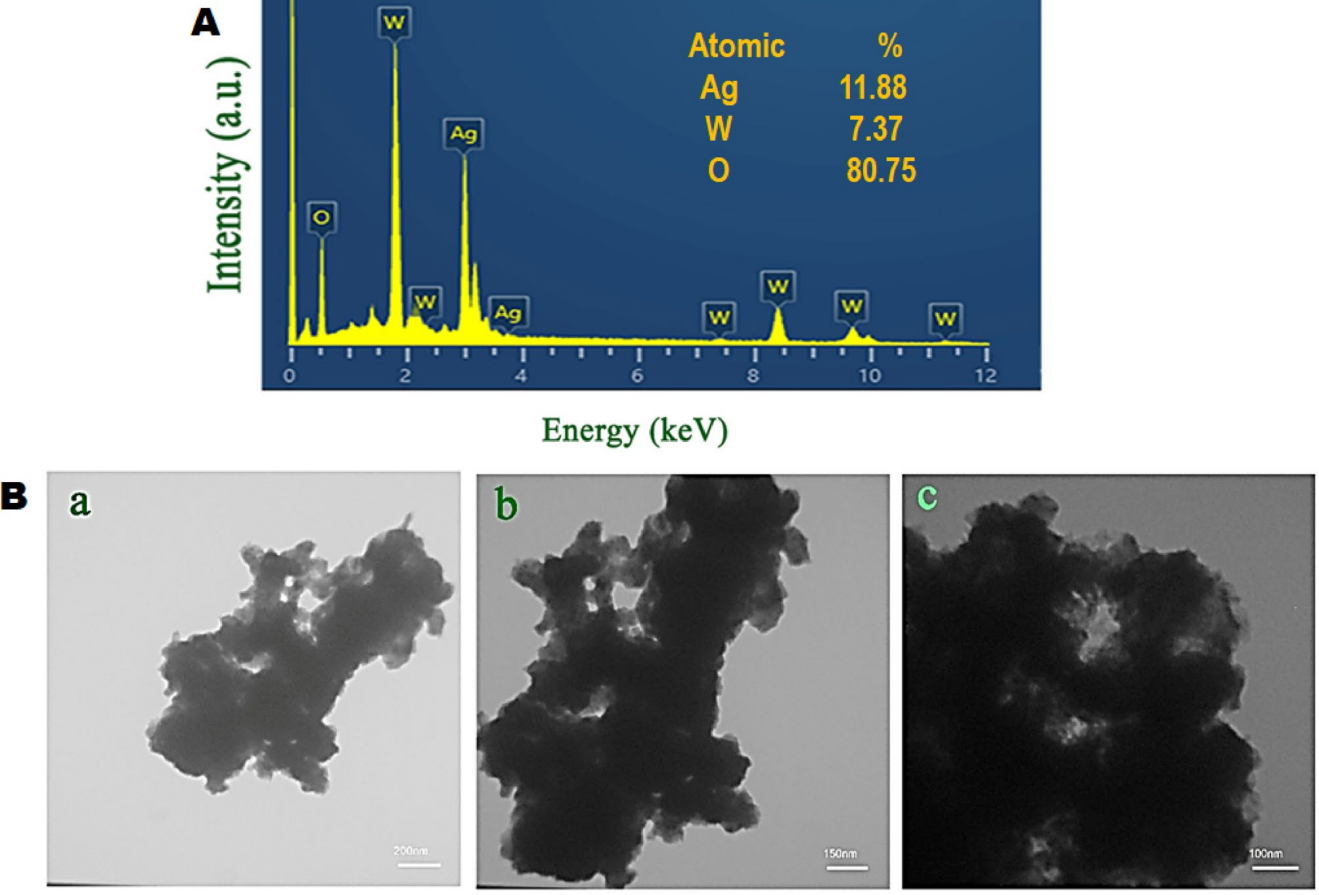
(**A**) EDS spectrum and (**B**) TEM images of Ag_2_WO_4_ nanoparticles.

EDS analysis was also performed to explore the elemental composition of the sample, the spectrum of which is displayed in Fig. 2A. The detection of only silver, tungsten, and oxygen elements in the prepared sample pattern is evidence of the formation of pure silver tungstate via the sucrose-assisted combustion approach, which is a good verification of the XRD results.

The internal morphology of the sucrose-assisted synthesized powders was checked by taking TEM images at different magnifications (see Fig. 2B). By assembling the sphere-like nanoparticles together, a silver tungstate cluster-like nanostructure is created according to the TEM images, which is a good endorsement of the FESEM outcomes.

### Electrochemical evaluation of the Ag₂WO₄/IL-modified electrode

**Fig. 3:**
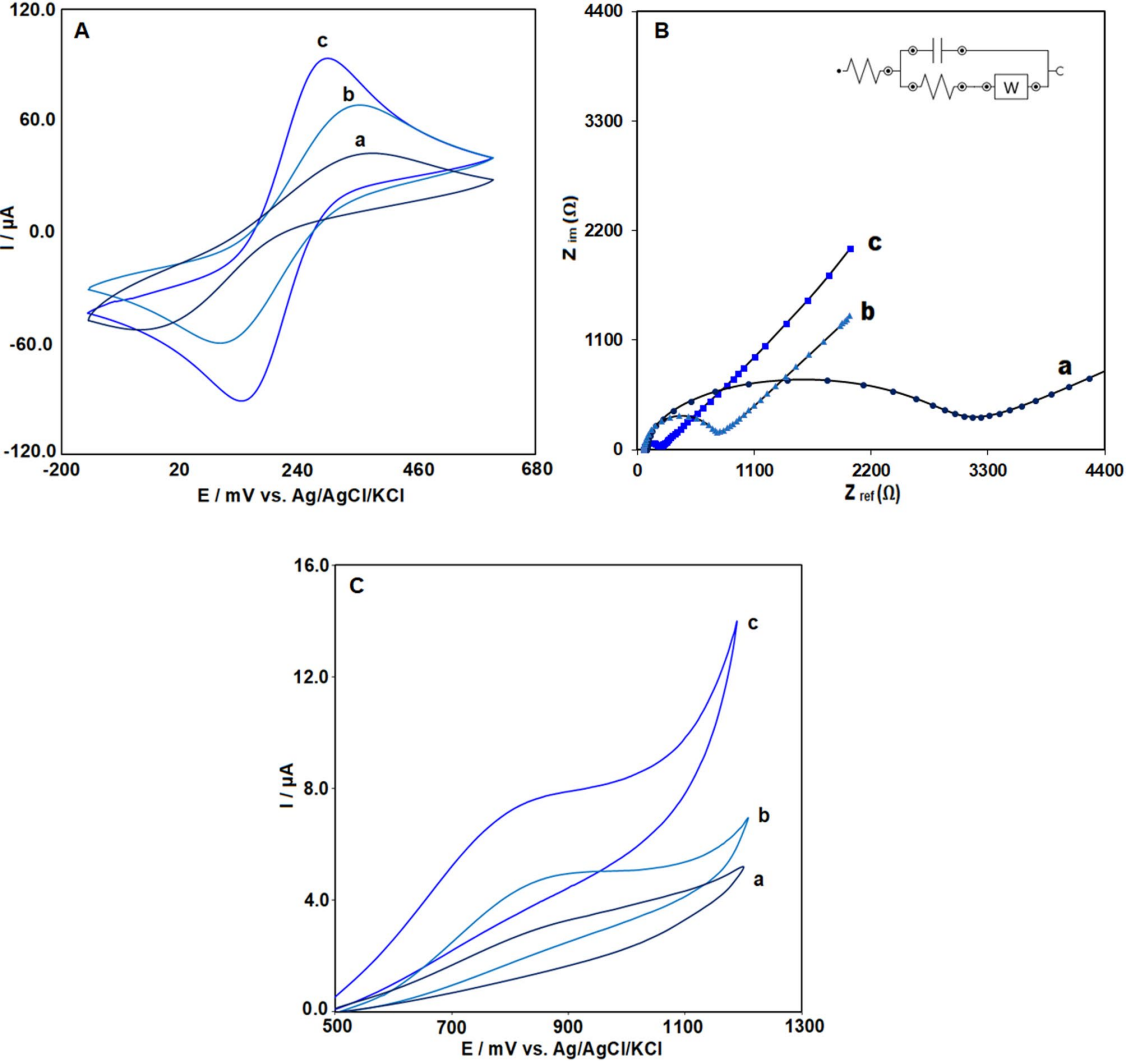
(**A**) Cyclic voltammograms and (**B**) Nyquist plots of the impedance spectra recorded in 5.0 mM [Fe(CN)₆]^3^⁻/^4^⁻ containing 0.1 M KCl, and (**C**) electrochemical response of 50 μM VRT recorded by CV in 0.1 M PBS (pH 7.0) at a scan rate of 50 mV s⁻^1^ for (**a**) CPE, (**b**) IL/CPE, and (**c**) Ag₂WO₄/IL/CPE electrodes.

To investigate the electrochemical properties of the Ag₂WO₄/IL/CPE, cyclic voltammetry (CV) was performed using 5 mM K₃Fe(CN)₆ dissolved in 0.1 M KCl as the redox probe. Figure 3A presents the cyclic voltammograms of the bare CPE (a), IL/CPE (b), and Ag₂WO₄/IL/CPE recorded at a scan rate of 50 mV/s. At the bare electrode (bare CPE), weak but distinguishable oxidation and reduction peaks were seen. The observed enhancement in current intensity after the addition of the ionic liquid is ascribed to its suitable ionic conductivity and its role in improving the electrochemical environment of the modified electrode. Ionic liquids typically improve the charge transfer at the electrode surface by increasing the ion mobility, ultimately leading to an improved electrochemical signal. In the next step, the addition of Ag₂WO₄ nanoparticles (NPs) to the electrode surface further increased the current intensity compared to the other two electrodes. This can be explained by the semiconducting properties of Ag₂WO₄, which facilitate faster electron transfer at the electrode interface. The synergistic effect between the ionic liquid and Ag₂WO₄ NPs likely contributes to the improved electrochemical performance, as Ag₂WO₄ enhances the active surface area and provides more sites for redox reactions, further boosting the current response.

To analyze the active surface area of the sensing material, CV was employed alongside the Randles–Sevcik equation Fig. S1. The study of the irreversible process was carried out at 298 K, utilizing 0.5 mM K₃Fe(CN)₆ as the analyte in a 0.1 M KCl, with measurements performed at multiple scan rates (10–200 mV/s). The equation used is as follows^32^:

1 $${\mathrm{I}}_{{\mathrm{p}}} = \, \left( {{2}.{69 } \times { 1}0^{{5}} } \right){\text{ n}}^{{{3}/{2}}} {\text{A C}}_{0} {\mathrm{D}}^{{{1}/{2}}} v^{{{1}/{2}}}$$

As shown in Eq. (1), n corresponds to the number of electrons transferred during the electrode process, I_p_ denotes the peak current, and *ν* corresponds to the scan rate. The concentration of the K₃[Fe(CN)₆] (0.5 mM) is indicated by C_0_, while the electrode surface area (A) is calculated from the slope of the linear plot of peak current (I_p_) versus the square root of the scan rate (*ν*^1/2^). The diffusion coefficient (D_0_) is given as 7.6 × 10^–6^ cm^2^ s^−1^. The surface area of the bare CPE was determined to be 0.067 cm^2^, while that of the Ag₂WO₄/IL/CPE increased significantly to 0.138 cm^2^. Notably, the modified electrode exhibits a substantially larger active surface area compared to the bare CPE.

EIS measurements were carried out in a solution comprising the [Fe(CN)₆]^3^⁻/^4^⁻ redox system with 0.1 M KCl. Figure 3B presents the equivalent electrical circuit used to fit the Nyquist plots for the bare carbon paste electrode (CPE), IL-modified CPE, and Ag₂WO₄/IL-modified CPE. The bare CPE exhibited a charge-transfer resistance (R_ct_) of 3 KΩ, indicating relatively slow electron transfer at the electrode interface. Modification with the ionic liquid (IL) decreased R_ct_ to 499 Ω, reflecting the enhanced conductivity imparted by the IL within the carbon paste matrix. The Ag₂WO₄/IL-modified CPE showed a significantly smaller semicircle in the Nyquist plot, corresponding to a further reduced R_ct_ of approximately 159 Ω. The observed pronounced decrease can be ascribed to the synergistic effect arising from the combined action of Ag₂WO₄ nanoparticles and the ionic liquid, which together facilitate faster electron transfer and improve the electrical conductivity at the electrode surface.

The CV responses of 40 μM VRT at pH 7.0 (0.1 M PBS) and a scan rate of 50 mV/s were examined using bare CPE, IL/CPE, and Ag₂WO₄/IL/CPE electrodes (Fig. 3C). A stepwise increase in current intensity was observed with each successive electrode modification. At bare CPE, the current was low due to moderate conductivity (2.9 µA). The addition of ionic liquid (IL) to form IL/CPE improved conductivity and charge transfer, increasing the current (4.8 µA). The highest current (7.7 µA) was observed at Ag₂WO₄/IL/CPE, which also showed a reduction in oxidation potential of VRT. This was due to the high conductivity and semiconducting properties of Ag₂WO₄, which enhanced electron transfer and provided more active sites. The synergistic effect between Ag₂WO₄ and IL led to the best electrochemical response.

### Study of pH

**Fig. 4:**
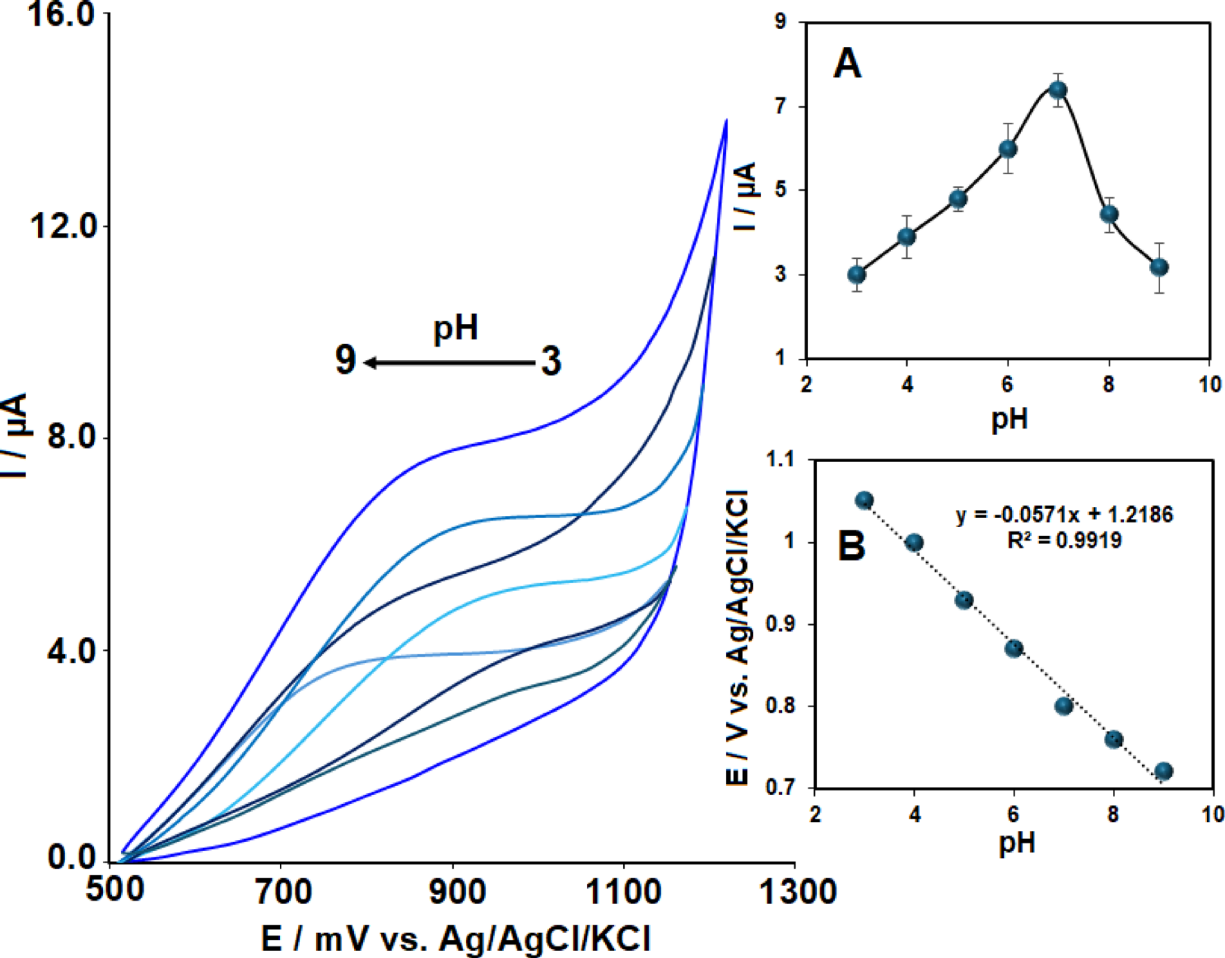
Voltammograms of 40 µM VRT recorded at Ag₂WO₄/IL/CPE in 0.1 M PBS at pH values ranging from 3 to 9. Insets show the dependence of (**A**) anodic peak current (Iₐₚ) and (**B**) anodic peak potential (Eₐₚ) on pH.

Effect of pH on the electrochemical oxidation of 40 μM VRT at the Ag₂WO₄/IL/CPE was evaluated by varying the pH values (Fig. 4). An incremental rise in oxidation peak current was observed at the modified electrode as the pH increased from 3.0 to 7.0, after which a gradual decrease occurred at pH values above 7.0. The relationship between peak current and peak potential with respect to pH is illustrated in the insets of Fig. 4. These results indicate that the highest current response occurs at pH 7, with reduced currents observed at both higher and lower pH levels (Inset A of Fig. 4). Therefore, based on the maximum peak current observed at pH 7, this pH was selected as the optimal pH in this study. Additionally, a peak potential vs. pH curve was constructed, and the corresponding linear equation was derived (Inset B). The slope of the curve was found to be 57 mV/pH, which closely matches the theoretical value of 59 mV/pH, implying equal involvement of protons and electrons in the reaction.

### Scan rate effect

**Fig. 5:**
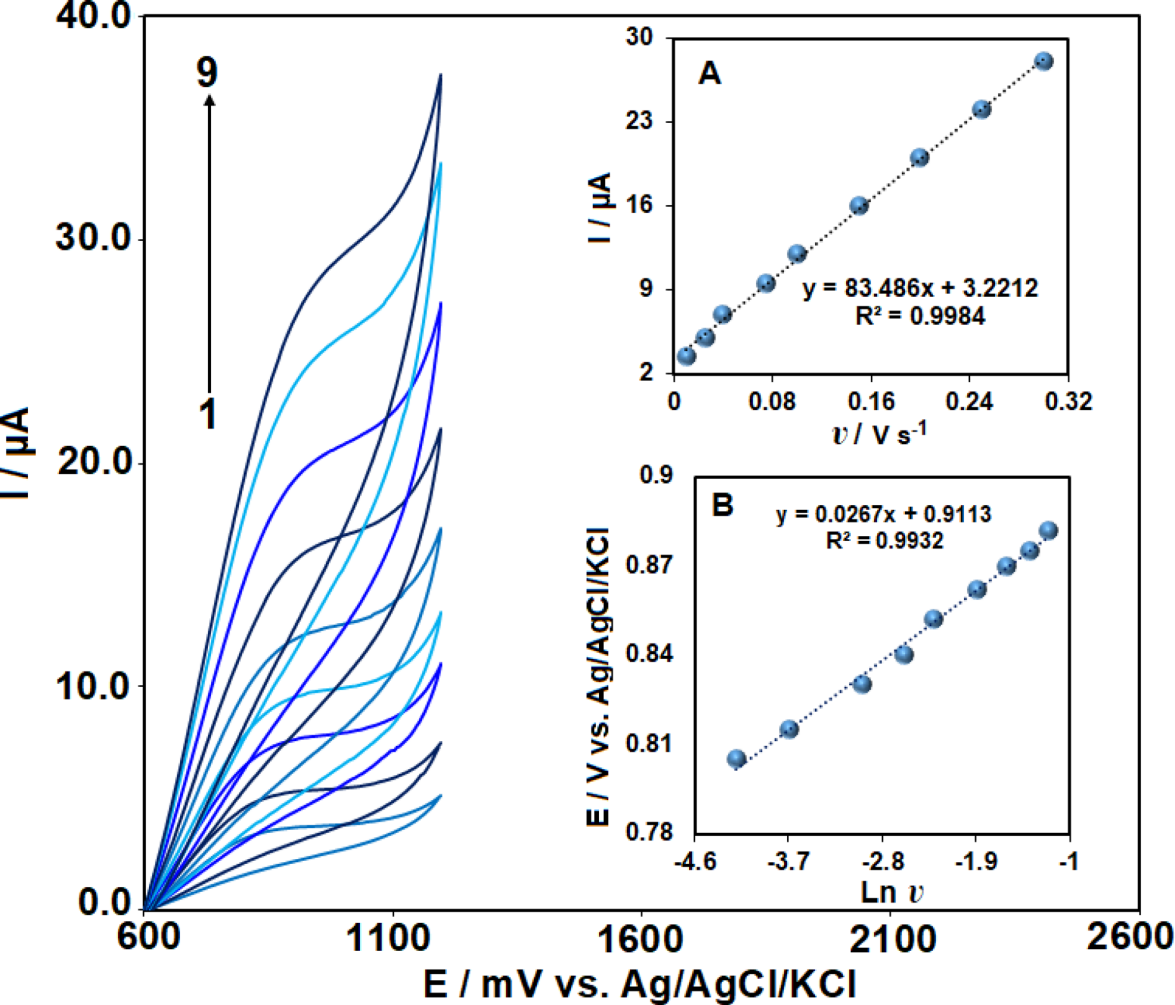
Cyclic voltammograms of Ag₂WO₄/IL/CPE recorded at scan rates from 15 to 300 mV/s (1–9) in 0.1 M PBS containing 40 μM VRT. Inset: (**A**) Dependence of anodic peak current on scan rate. (**B**) Plot of anodic peak potential (Eₐₚ) versus ln ʋ.

The effect of scan rate on the charge transport behavior of the Ag₂WO₄/IL/CPE sensor in the presence of 50 μM VRT was investigated using cyclic voltammetry in 0.1 M PBS (pH = 7.0) across a range of scan rates from 15 to 300 mV/s. The anodic peak current increased steadily with scan rate, as shown in Fig. 5. Moreover, as depicted in Fig. 5 (inset A), a linear correlation was observed between the anodic peak current and the scan rate. A linear regression of I (µA) = 83.48 *v* (V/s) + 3.2 (R^2^ = 0.998) confirms that vortioxetine oxidation at the modified electrode follows an adsorption-controlled process.

Using Laviron’s equation, the electron transfer number (n) for vortioxetine oxidation at the electrode surface was estimated.

$${E}_{p}={E}^{0}+\left(\frac{RT}{\alpha nF}\right)\mathit{ln}\left(\frac{RT{k}^{0}}{\alpha nF}\right)+\left(\frac{RT}{\alpha nF}\right)ln v$$

The standard heterogeneous rate constant is represented by k∘, the scan rate by *v*, and the number of electrons transferred by n. The formal redox potential is denoted as E∘, while α corresponds to the charge transfer coefficient. From the slope of E_pa_ versus ln *v*, the value of αn was calculated to be 0.96 (Inset B). In irreversible electrode processes, the charge transfer coefficient (α) is commonly assumed to have a value of 0.5. Therefore, the number of electrons exchanged in the electro-oxidation of VRT was determined to be 1.91 (approximately 2). These results suggest that the electro-oxidation of VRT at the Ag₂WO₄/IL/CPE surface proceeds via a two-electron, two-proton transfer mechanism.

**Fig. 6:**
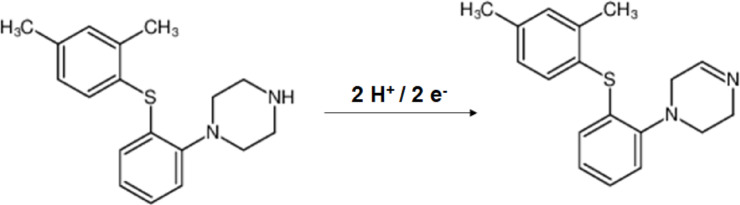
Proposed electro-oxidation mechanism of VRT at the surface of the Ag₂WO₄/IL/CPE.

Based on the electrochemical behavior of vortioxetine, the oxidation mechanism involving the loss of two electrons and two protons appears to be the more plausible pathway, especially at physiological pH (around 7). At this pH, the environment is sufficiently neutral to favor the deprotonation of the amine group, enabling a two-proton, two-electron transfer process. This mechanism is consistent with the observed redox characteristics of similar tertiary amines and aligns with the chemical structure of vortioxetine, where the nitrogen atom can act as a key site for electron and proton loss (Fig. 6). Therefore, the two-electron/two-proton oxidation pathway is likely to dominate under near-neutral conditions.

### Optimization of electrochemical determination

**Fig. 7:**
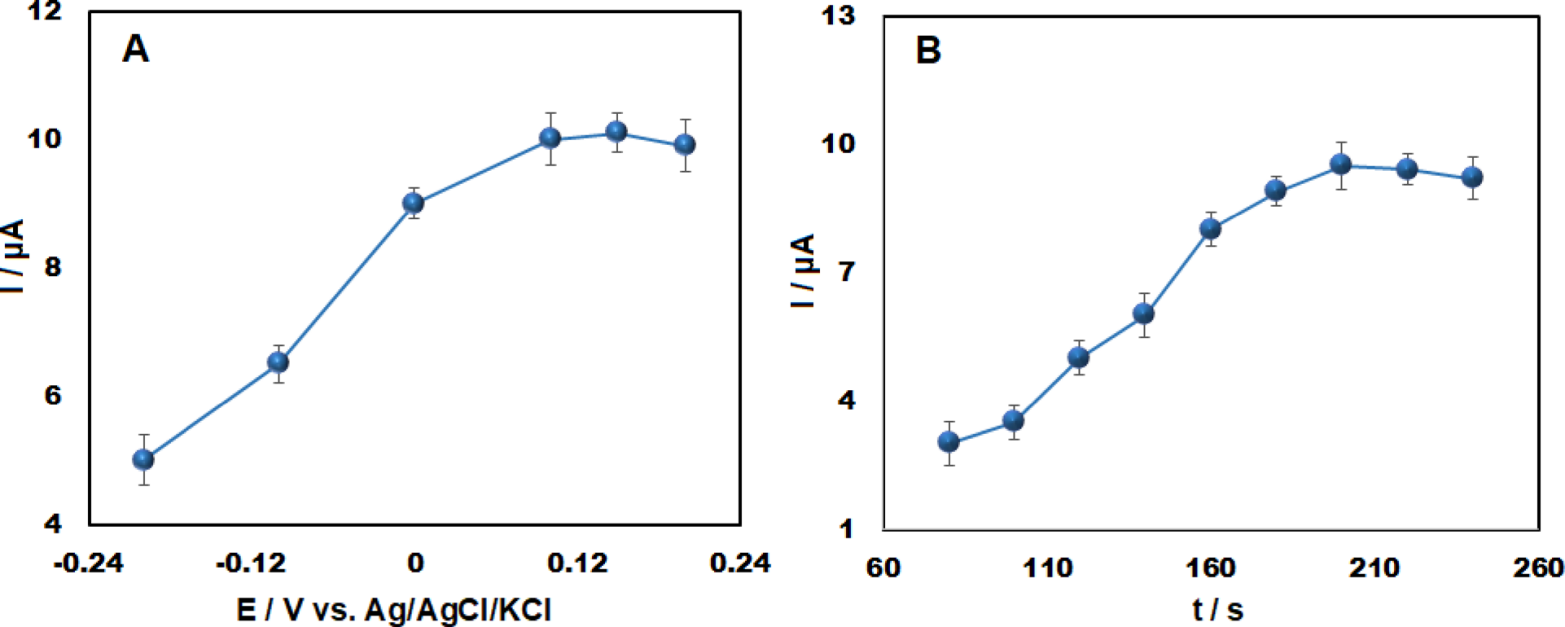
Electrochemical response of 50 μM VRT at Ag₂WO₄/IL/CPE as a function of (**A**) accumulation potential and (**B**) accumulation time (n = 3).

Using AdsDPV at the Ag₂WO₄/IL/CPE electrode, the influence of accumulation time (t_ac_) and accumulation potential (E_ac_) on the electrochemical response of VRT was investigated. The influence of accumulation potential from -0.2 to + 0.2 V was studied (Fig. 7A). The oxidation peak current progressively increased with rising potential, attaining its maximum at + 0.1 V. An increase in potential beyond + 0.1 V caused a gradual reduction in the peak current. Therefore, an accumulation potential of 0.1 V was selected for drug preconcentration.

The influence of accumulation time (tₐ_c_) on the modified electrode was investigated by maintaining a constant accumulation potential (Eₐ_cc_) over a range of 80 to 240 s. The voltammetric response progressively increased with accumulation time, reaching its maximum adsorption capacity at 200 s on the Ag₂WO₄/IL/CPE surface (Fig. 7B). Consequently, an E_ac_ of 0.1 V and t_ac_ of 200 s were selected as the optimal conditions for the preconcentration step in the electrochemical analysis of VRT.

### Calibration curve

**Fig. 8:**
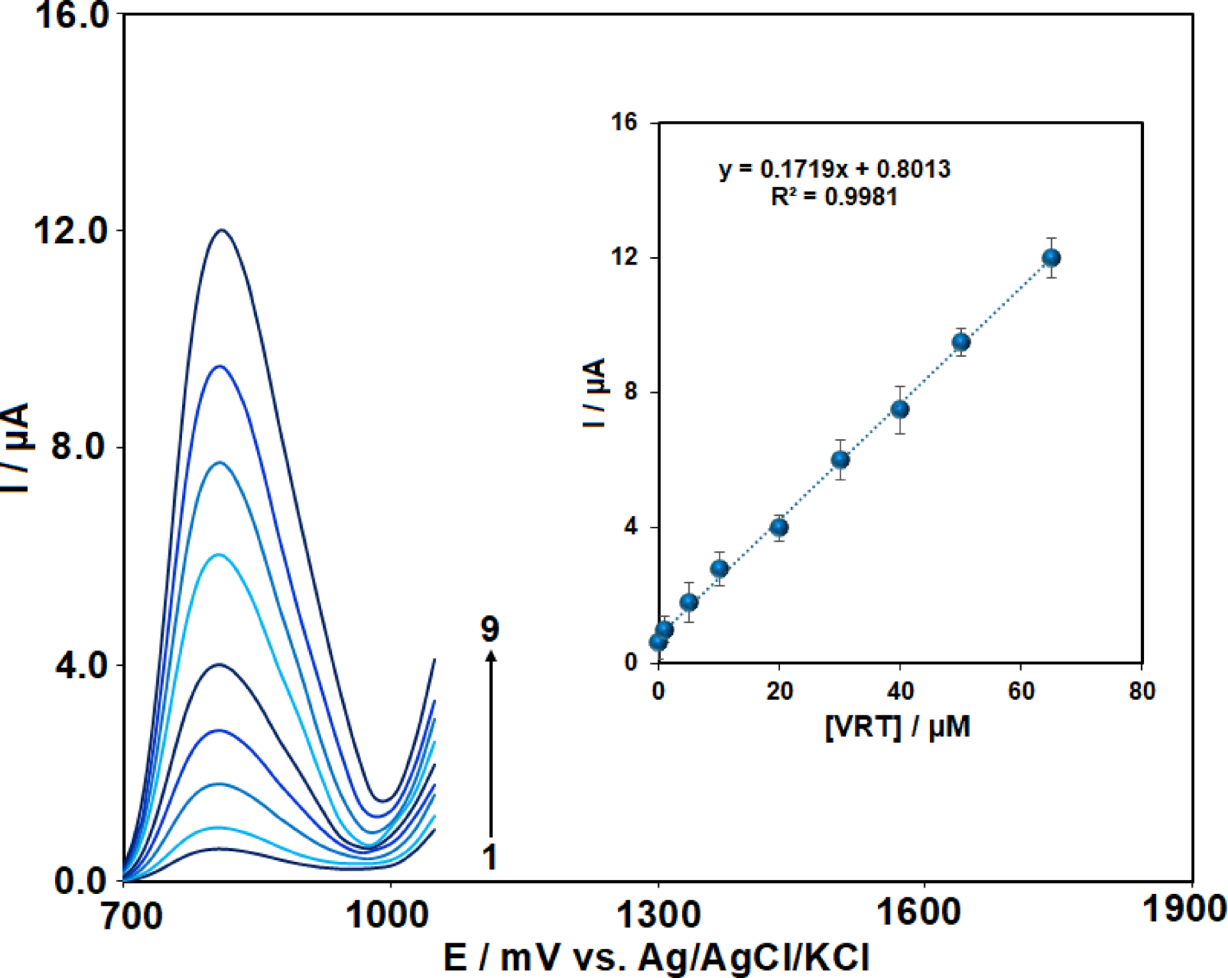
AdsDPV voltammograms obtained for VRT at concentrations ranging from 0.03 to 65 μM (curves 1–9) in 0.1 M PBS using Ag₂WO₄/IL/CPE. The inset shows the corresponding calibration plot of peak current versus VRT concentration (n = 3).

To evaluate the method’s practical applicability, the oxidative peak current was analyzed as a function of VRT concentration using AdsDPV at the Ag₂WO₄/IL/CPE. Figure 8 demonstrates that the peak current response increased proportionally with increasing concentration, exhibiting linearity over the concentration range of 0.03 to 65 µM. This correlation follows the linear regression equation: y = 0.1719x + 0.8013; (R^2^ = 0.998).

LOD and limit of quantification (LOQ) were estimated as 3.3 and 10 times the ratio of the blank standard deviation (S_b_) to the calibration slope (m), respectively. Accordingly, the LOD was calculated to be 0.01 µM, while the LOQ was determined as 0.03 µM.

The analytical performance of the proposed Ag₂WO₄/IL/CPE sensor for VRT detection was compared with previously reported electrochemical sensors (Table 1). The developed electrode exhibits a wide linear range (0.03–60 µM) and a low detection limit (0.01 µM), comparable to or better than many earlier systems. In addition to its analytical performance, the sensor shows excellent selectivity against common interferents and provides stable and reproducible responses in both tablet and urine samples, demonstrating strong real-sample applicability. Importantly, the fabrication process is simple, cost-effective, and based on a green synthesis route, offering an environmentally friendly alternative to electrodes involving complex nanocomposite preparations or noble metal deposition. These combined advantages highlight the potential of the Ag₂WO₄/IL/CPE electrode as a practical and sustainable platform for routine electrochemical determination of VRT.

**Table 1:** Comparison of the linear range and LOD of the Ag₂WO₄/IL/CPE sensor with other reported electrochemical sensors.

Electrode	Linear range (µM)	LOD (µM)	Sample	Ref
AuNPs/GRP/GCE	0.1–6.0	0.05	Tablet	^33^
Boron-doped diamond electrode	0.009–0.148	0.002	Blood rat serum	^34^
GPE	0.05–50.0	0.02	Plasma and urine	^35^
CNTs-SP	0.03–5	0.01	Tablet and urine	^36^
eCNF/CNT/NiCo-GCE	0.01–3.0	0.001	Tablets, urine, and plasma	^37^
Ag_2_WO₄/IL/CPE	0.03–60.0	0.01	Tablet and urine	This study

### Repeatability, reproducibility, and stability of Ag_2_WO₄/IL-modified CPE

To evaluate repeatability, five successive AdsDPV measurements were recorded with the same fabricated Ag_2_WO₄/IL/CPE in a 50.0 μM VRT. The sensor exhibited good repeatability with an RSD of 3.7% (Fig. S2A). To assess the reproducibility of the electrode fabrication, three identically prepared modified electrodes were used to measure a 50 μM of Vortioxetine (Fig. S2B). The RSD was found to be 4.2%, demonstrating satisfactory reproducibility in the electrode fabrication process. Moreover, Ag_2_WO₄/IL/CPE was kept at room temperature in the laboratory, and after 4 weeks, a measurement of 50 μM Vortioxetine was performed (Fig. S2C). The results showed that the electrode retained 95.8% of its initial signal, indicating excellent long-term stability and durability of the sensor under standard laboratory storage conditions.

### Selectivity investigation

**Fig. 9:**
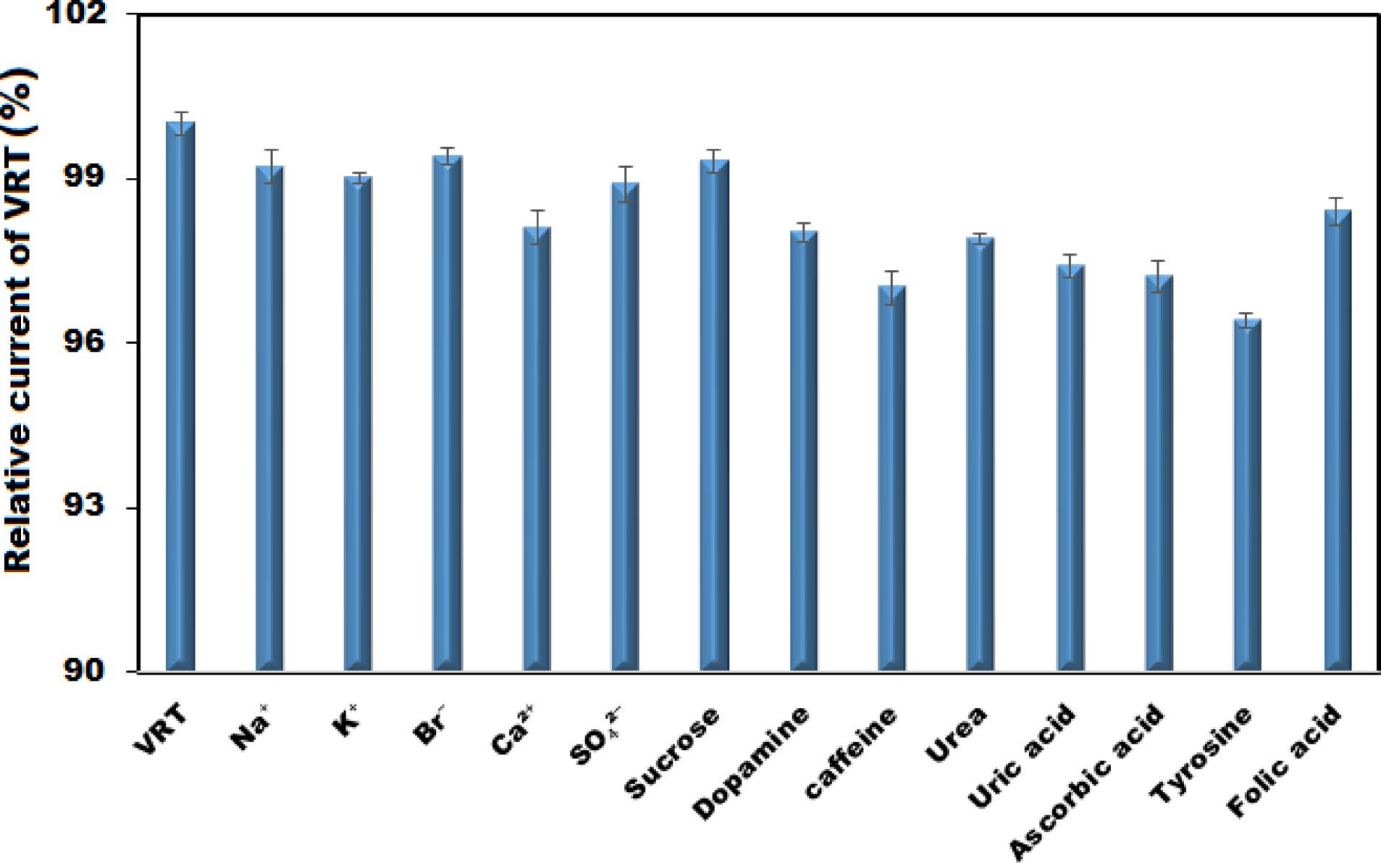
Influence of several potential interfering species on the electrochemical response of 10 μM VRT at Ag₂WO₄/IL/CPE in 0.1 M PBS (pH 7.0).

The selectivity of the proposed electrode was assessed by examining its response in the presence of potential interfering substances. The results demonstrated that the addition of 50-fold excess concentrations of common biological and pharmaceutical compounds such as sucrose, folic acid, dopamine, urea, and ascorbic acid, tyrosine, caffeine and uric acid as well as 25-fold excess concentrations of inorganic ions including Na⁺, K⁺, Ca^2^⁺, Br⁻, and SO₄^2^⁻ resulted in less than 5% deviation in the voltammetric signal of 10 μM VRT (Fig. 9). This minimal signal disturbance indicates that the proposed sensor exhibits high selectivity and strong resistance to potential interferences, confirming its suitability for the accurate determination of VRT even in complex biological or pharmaceutical matrices.

### Tablet and urine sample analysis

The proposed sensor was evaluated using AdsDPV for the determination of VRT in practical clinical samples, including tablets and urine. The standard addition method was applied by spiking the samples with known concentrations of VRT: 0.0, 5.0, 10.0, and 15.0 μM for tablets, and 0.0, 10.0, 15.0, and 20.0 μM for urine. Sample preparations for VRT tablets and urine were carried out as described in Section “Real sample preparation”, and a summary of the results is presented in Table 2. The observed satisfactory recoveries and low RSD values confirm the reliability and accuracy of the developed sensor for VRT detection in biological matrices.

**Table 2:** Determination of VRT in real samples, including tablet and human urine, using the Ag₂WO₄/IL/CPE sensor.

Samples	Spiked (μM)	Found (μM)	Recovery (%)	R.S.D. (%)
Tablet	0.0	5.0	–	2.9
	5.0	9.7	97.0	2.4
	10.0	15.5	103.3	3.7
	15.0	20.5	102.5	2.9
Urine	0.0	N.D	–	–
	10.0	9.8	98.0	3.6
	15.0	15.4	102.6	3.0
	20.0	19.4	97.0	2.1

Furthermore, the results obtained from the fabricated electrode for VRT determination were compared with those from HPLC analysis (Table 3). At a 95% confidence interval, the calculated t-value was less than the critical t-value, indicating no significant difference between the two datasets and confirming the accuracy and reliability of the proposed sensor.

**Table 3:** Comparison of VRT determination in real samples using Ag₂WO₄/IL/CPE and HPLC methods.

Sample	Spiked (μM)	Found (μM)		t _exp_
		DPV	HPLC	
Tablet	0.0	5.04 ± 0.15	5.09 ± 0.28	0.27
	10.0	15.46 ± 0.57	14.92 ± 0.25	1.50
Urine	10.0	9.82 ± 0.35	10.03 ± 0.36	0.72
	20.0	19.39 ± 0.41	19.80 ± 0.46	1.15

## Conclusion

In present study, silver tungstate nanostructures were successfully synthesized via a simple and eco-friendly combustion method using sucrose. A simple, fast, and selective electrochemical method was developed for the determination of VRT using an Ag₂WO₄/IL modified carbon paste electrode (CPE). The synergistic properties of silver, tungsten, and the ionic liquid, along with the increased effective surface area of the modified electrode, significantly enhanced the sensor’s performance, resulting in high detection efficiency, a low LOD of 0.01 µM, and a wide linear detection range of 0.03–60 μM for Vortioxetine. The modified CPE exhibited high sensitivity and reliability, as confirmed by recovery tests that yielded consistent and accurate results. Furthermore, the Ag₂WO₄/IL/CPE demonstrated excellent reproducibility and repeatability, highlighting its potential as an efficient platform for biomedical and clinical applications.

## Supplementary Information

Below is the link to the electronic supplementary material.

Supplementary Material 1

## Data Availability

The datasets used and analysed during the current study are available from the corresponding author on reasonable request.
