# Supplementary material for: Green synthesis of silver tungstate/ionic liquid-modified electrode for highly efficient electrochemical detection of the antidepressant Vortioxetine
Source: Sci Rep. 2025 Dec 5;15:45772. doi: 10.1038/s41598-025-28420-9 (PMC12756288; doi:10.1038/s41598-025-28420-9)
Supplement: Supplementary file 1 — Supplementary Material 1 [file 41598_2025_28420_MOESM1_ESM.docx]

**Green synthesis of silver tungstate/ionic liquid-modified electrode for highly efficient electrochemical detection of the antidepressant Vortioxetine**

Sahar Zinatloo-Ajabshir^a^, Hamid Akbari Javar^b^, Hadi Mahmoudi-Moghaddam^c,*^, Ali Azari^d^

*^a^Department of Chemical Engineering, University of Bonab, P.O. Box. 5551395133, Bonab, Iran*

*^b^ Pharmaceutics Department, Faculty of Pharmacy, Tehran University of Medical Sciences, Tehran, Iran*

^c^ *Pharmaceutics Research Center, Institute of Pharmaceutical Sciences, Kerman University of Medical Sciences, Kerman, Iran*

*^d^Environmental Health Engineering, School of Public Health, Qom University of Medical Sciences, Qom, Iran.*

[*h.mahmoudi@kmu.ac.ir*](mailto:h.mahmoudi@kmu.ac.ir)

**Electrochemical parameters**

The electrochemical parameters were optimized using *NOVA 2.1* software. The optimal accumulation potential and time were set at 0.1 V and 200 s, respectively. For DPV measurements, a step potential of 0.0044 V, modulation amplitude of 0.05 V, and modulation time of 0.04 s were selected to maintain a scan rate of 0.05 V/s, ensuring stable and reproducible peak currents.

**HPLC analysis**

HPLC analyses were performed on an Agilent 1260 system equipped with a DAD set at 225 nm. A RP-18 column (3.5 µm, 100 × 4.6 mm) was used with an isocratic mobile phase of phosphate buffer and methanol (30:70, v/v) at a flow rate of 1.3 mL·min⁻¹ and a column temperature of 50 °C. The injection volume was 20 µL, and working solutions and mobile phases were prepared daily.


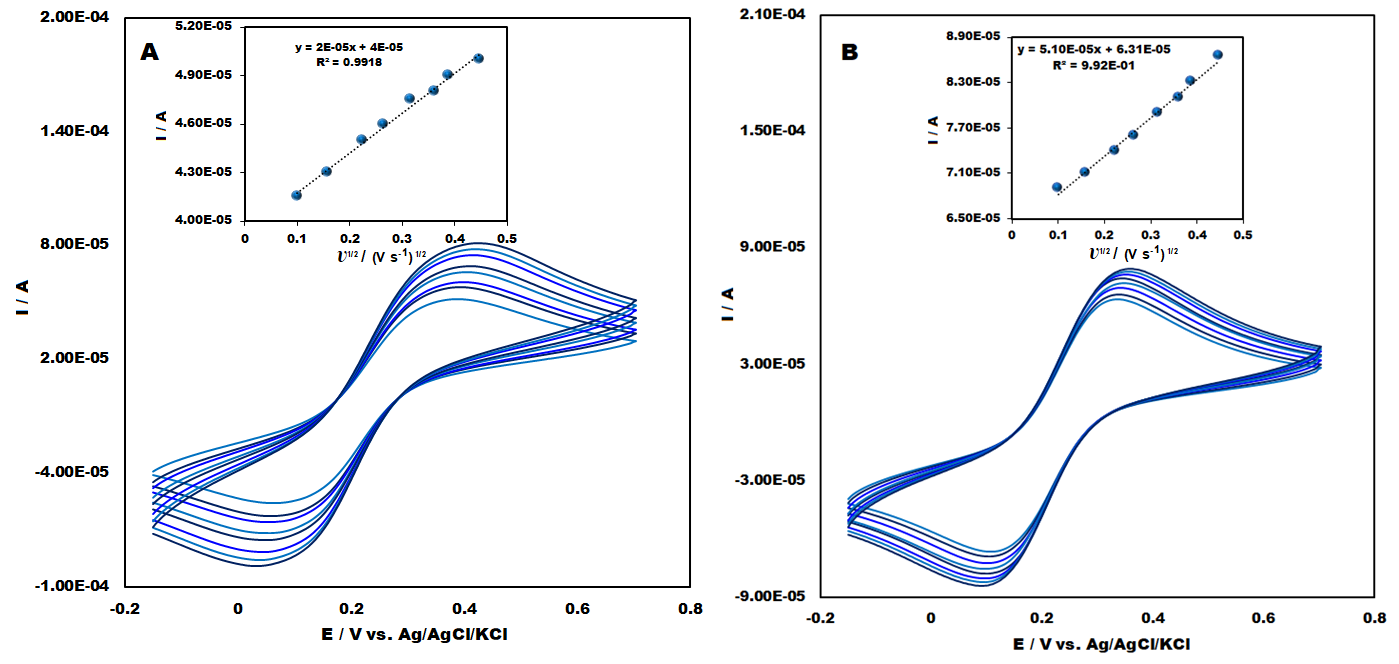


Fig. S1. Cyclic voltammograms of (A) bare CPE and (B) Ag₂WO₄/IL/CPE recorded in 0.5 mM [Fe(CN)₆]³⁻/⁴⁻ containing 0.1 M KCl at various scan rates ranging from 0.01 to 0.20 V/s.


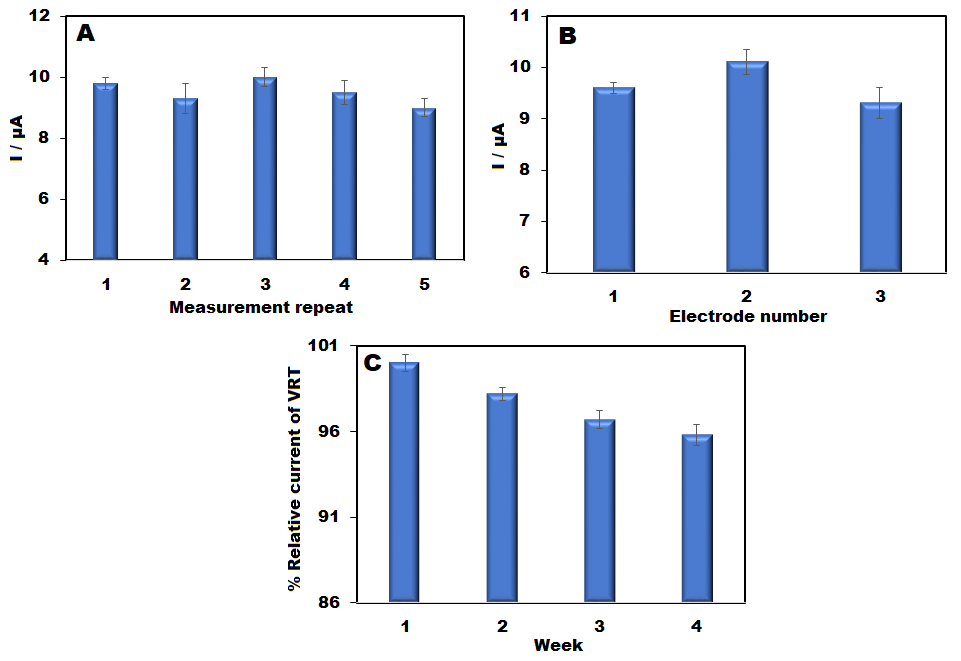


**Fig. S2.** Electrochemical performance of 50 µM VRT at the Ag₂WO₄/IL-modified CPE: (A) repeatability evaluated by five consecutive measurements using a single electrode, (B) reproducibility across three independent electrodes, and (C) stability monitored over four weeks.
